# A metagenomic insight into the Yangtze finless porpoise virome

**DOI:** 10.3389/fvets.2022.922623

**Published:** 2022-09-02

**Authors:** Zhigang Liu, Xin Ding, Muhammad Shahan Haider, Farah Ali, Han Yu, Xin Chen, Shuaishuai Tan, Yuan Zu, Wenlong Liu, Bangzhi Ding, Aifang Zheng, Jinsong Zheng, Zhengyi Qian, Hassan Ashfaq, Daoping Yu, Kun Li

**Affiliations:** ^1^College of Life Science, Anqing Normal University, Anqing, China; ^2^Research Center of Aquatic Organism Conservation and Water Ecosystem Restoration in Anhui Province, Anqing Normal University, Anqing, China; ^3^Bahria University Medical and Dental College Karachi, Karachi, Pakistan; ^4^Department of Theriogenology, Faculty of Veterinary and Animal Sciences, The Islamia University of Bahawalpur, Bahawalpur, Pakistan; ^5^Institute of Hydrobiology, Chinese Academy of Sciences, Beijing, China; ^6^Hubei Yangtze River Ecological Protection Foundation, Wuhan, China; ^7^Institute of Continuing Education and Extension, University of Veterinary Animal Sciences, Lahore, Pakistan; ^8^Institute of Traditional Chinese Veterinary Medicine, College of Veterinary Medicine, Nanjing Agricultural University, Nanjing, China; ^9^MOE Joint International Research Laboratory of Animal Health and Food Safety, Nanjing Agricultural University, Nanjing, China

**Keywords:** Yangtze finless porpoise, diversity, virus, metagenomics, *Arenaviridae*

## Abstract

The Yangtze finless porpoise (*Neophocaena phocaenoides asiaeorientalis*) inhabiting the Yantze River, China is critically endangered because of the influences of infectious disease, human activity, and water contamination. Viral diseases are one of the crucial factors that threatening the health of Yangtze finless porpoise. However, there are few studies which elaborate the viral diversity of Yangtze finless. Therefore, this study was performed to investigate the viral diversity of Yangtze finless by metagenomics. Results indicated that a total of 12,686,252 high-quality valid sequences were acquired and 2,172 virus reads were recognized. Additionally, we also obtained a total of 10,600 contigs. Phages was the most abundant virus in the samples and the ratio of DNA and RNA viruses were 69.75 and 30.25%, respectively. *Arenaviridae, Ackermannviridae* and *Siphoviridae* were the three most predominant families in all the samples. Moreover, the majority of viral genus were *Mammarenavirus, Limestonevirus* and *Lambdavirus*. The results of gene prediction indicated that these viruses play vital roles in biological process, cellular component, molecular function, and disease. To the best of our knowledge, this is the first report on the viral diversity of Yangtze finless porpoise, which filled the gaps in its viral information. Meanwhile, this study can also provide a theoretical basis for the establishment of the prevention and protection system for virus disease of Yangtze finless porpoise.

## Introduction

*Neophocaena asiaeorientalis* ssp. *asiaeorientalis* also known as Yangtze finless porpoises (YFP) are fresh-water mammals that inhabit Yangtze River in People's Republic of China. They are critically endangered species with an estimated decreasing population of 500–1,800 (1). The survival of Yangtze finless porpoises is affected by multiple factors such as human activities, industrial development, mining, shipping, fishing, dams, water pollution and climate change. Efforts have been put into the research and conservation of this specie by protecting the areas, reserves and *in situ* conservation. Notably, the quantity of Yangtze finless porpoises is still gradually decreasing due to human activities over-fishing and water pollution of the Yangtze River. Water pollution is not only due to imbalance of the Yangtze River ecosystem but also caused the outbreak of viral and bacterial diseases, which posed a serious threat to the survival of Yangtze finless porpoises (2). Statistically, the population of Yangtze finless porpoises is <2,000 and it has been considered as the endangered species by the International Union for Conservation of Nature (IUCN) (3).

Viruses are one of the most abundant biological beings on the earth which are characterized by genomic flexibility, rapid transition, and smaller volume (4, 5). Viral habitat and diversity are important to formulate the prevention, control, and preparedness in case of emergency outbreaks (6). Moreover, this information is important to develop targeted drugs and vaccines against such diseases (6–8). The traditional virus identification/ detection methods include growth on cell culture and electron microscopy, serology, and molecular biology with each having its own benefits and limitations (9, 10). Recently, the high throughput sequencing-based techniques e.g., the metagenomics, has provided an important platform for the large-scale identification of known and novel viruses in a habitat (11, 12). A large amount of known and novel viruses has been identified from the pigs, ducks, turkey, pigeon, and bats by using viral metagenomics (13–17). However, little is known about the viral diversity in Yangtze finless porpoises. Therefore, the objective of this study was to investigate the viral diversity in Yangtze finless porpoise by metagenomics.

## Materials and methods

### Sample collection and processing

A total of 40 samples including heart, liver, spleen, lung, kidney, pancreas, lymph nodes and skin were collected from the dead Yangtze finless porpoises. The samples were collected from 2017 to 2019. Moreover, these samples were collected from 4 wild finless porpoises inhabiting the Yangtze River, aged 2–5, from the same site. The achieved samples were immediately stored in sterile plastic containers and transported to the laboratory and later stored at −80°C for further processing.

### Nucleic acid extraction and library construction

First, the tissue samples were mixed and homogenized and then the debris of the tissues and cells were removed by low-speed centrifugation. Additionally, multiple virologic separation methods including filtration and ultracentrifugation were used for purifying and concentrating the virus-like particle (VLP) in the solution. The nucleic acid of the obtained VLP was extracted by the method of virus DNA (dsDNA, ssDNA) and RNA (ssRNA, dsRNA) co-extraction using viral DNA/RNA extraction kit (Beijing Quanshijin Biotechnology Co., LTD). Afterwards, the genomic libraries were constructed based on viral genome types. The DNA library of dsDNA genome was constructed directly, whereas ssDNA needs to be synthesized into double-stranded DNA before DNA library construction. Prior to the RNA library construction, the RNA genome needs to be converted into double-stranded cDNA through reverse transcription and two-strand synthesis. The total amount and purity of the DNA and RNA were evaluated, and the unqualified nucleic acids need to be re-extracted or undergo whole-genome amplification and purification. The qualified or amplified DNA samples were randomly shattered by an ultrasonic disruptor and the obtained short fragments of DNA were used for sequencing library construction. The final qualified libraries were used for high-throughput sequencing on the Illumina platform at Guangdong Meige Gene Technology Co., Ltd., China.

### Bioinformatics analysis

After obtaining the metagenomic sequencing data, the quality of data was evaluated using Soapnuke software to ensure the credibility of subsequent analysis results. The paired reads with adapter and low quality were discarded. Moreover, duplicate reads generated by PCR amplification were also required to be removed. The finally obtained high-quality sequences were used for further data analysis. The clean reads were aligned to the specific host genome to remove the host sequence by using SOAPaligner (v2.0.5) and BWA software's (v0.7.17). Additionally, the high-quality sequences were required to compare with the virus database to obtain the virus composition information in the sample. Meanwhile, the NCBI taxonomy database was used to count the virus taxonomy annotation information. The obtained high-quality reads of each sample were assembled to achieve a longer contigs sequence by using Megahit, Trinity and IDBA software. The quantity, length, and N50 of assembled sequences were counted and the high-quality reads were aligned to the assembled sequence to evaluate the utilization of assembled reads. The reads were compared with the identified viral contigs and the RPKM value of each contig was also calculated. The RPKM value was calculated with the following formula: RPKM% = [Contig reads/Total mapped reads × Contig length] × 100%. The MetaGeneMark was used to predict the gene sequences of viral contigs and assess the number and length of the predicted genes. The predicted gene protein sequences were compared with UniProtKB/Swiss-Prot database to obtain functional annotation information.

## Results

### Sequences analyses

**Figure 1:**
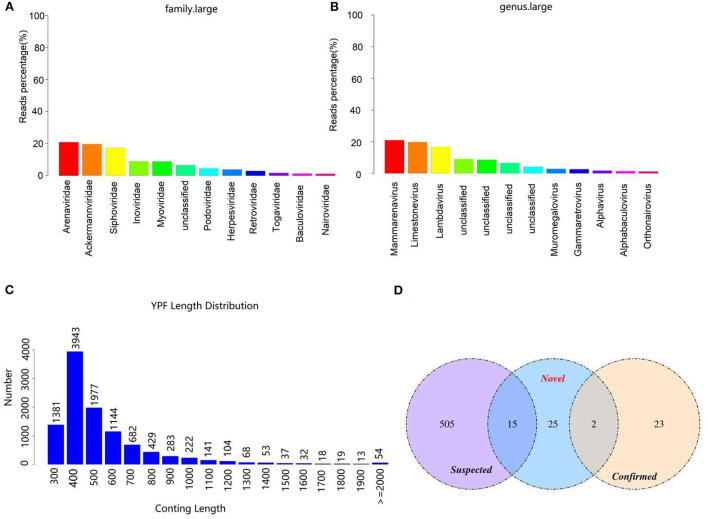
Viral composition and the length distribution of contigs. **(A)** The relative abundance of viral composition at the level of family. **(B)** The relative abundance of viral composition at the level of genus. **(C)** The length distribution of contigs. The abscissa represents the length of contigs and the ordinate indicates the number of contigs. **(D)** Comparison of the number of viruses contigs obtained by different methods.

After the filtration of raw data, a total of 12,686,252 high-quality sequences were obtained (Table 1). The obtained high-quality sequences were compared with the ribosomal database (Silva.132) and the host (*Neophocaena asiaeorientalis asiaeorientalis*) database and the corresponding sequences which were <80% of the total length of reads were removed to avoid the influences of ribosome and host sequence on subsequent analysis (Table 2). Additionally, the high-quality sequences were compared with the virus database to quickly obtain the virus composition information in the sample and a total of 2,172 virus reads were found (Supplementary Table S1). The virus classification information was statistically analyzed based on the annotation information of the NCBI taxonomy database. Statistical analysis indicated that *Phages* was the most abundant virus, accounting for approximately 62.94% of the total number of viruses (Supplementary Table S2). Meanwhile, the proportion of DNA and RNA viruses were 69.75% and 30.25%, respectively (Supplementary Table S3). The top 11 preeminent families in the collected samples at the family level are shown in Figure 1A. At the family level, *Arenaviridae, Ackermannviridae* and *Siphoviridae* were the three most predominant families, whereas *Inoviridae, Myoviridae, Podoviridae, Herpesviridae, Retroviridae, Togaviridae, Baculoviridae* and *Nairoviridae* were represented with a lower abundance. At the level of genus, *Mammarenavirus, Limestonevirus* and *Lambdavirus* were observed as the predominant. Furthermore, other genera including *unclassified_Inoviridae, unclassified_Myoviridae, unclassified_Podoviridae, Muromegalovirus, Gammaretrovirus, Alphavirus, Alphabaculovirus* and *Orthonairovirus* were indicated with a lower abundance (Figure 1B).

**Table 1:** Statistical analysis of the sequence information.

**Sample**	**Raw-base (G)**	**Raw-reads (PE)**	**Clean-base (G)**	**Clean-reads (PE)**	**Percent (%)**
YFP	14.35	47,824,812	3.81	12,686,252	26.53

**Table 2:** Statistical analysis of the sequence information after removing host sequence.

**Sample**	**Clean_reads (PE)**	**Rm_rRNA_clean (PE)**	**Rm_host_clean (PE)**
YFP	12,686,252	3,347,258(26.38%)	1,800,277(14.19%)

### Virus sequence identification

**Figure 2:**
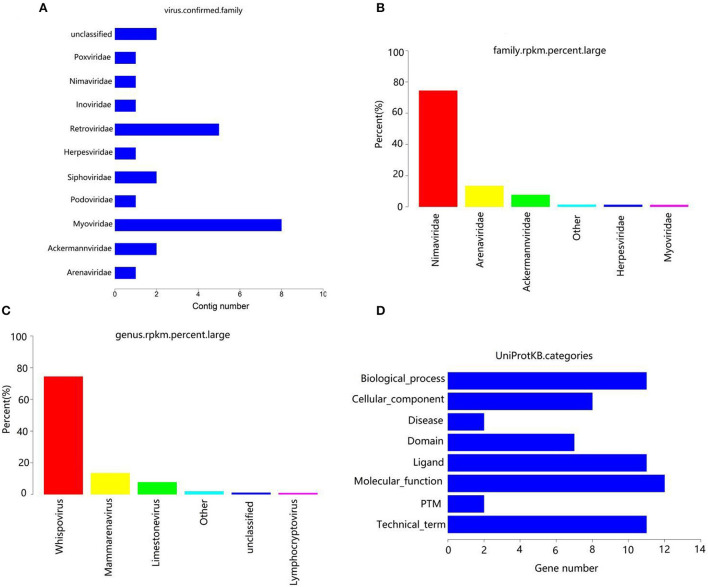
Annotation statistics of confirmed virus at the family level **(A)**. Abscissa: quantity of contrags; ordinates: annotation information. **(B)** RPKM percentage statistics maps at family level. **(C)** RPKM percentage statistics maps at genus level. **(D)** Uniprotkb category statistics.

After removing the host sequence, 10600 contigs were totally obtained (Table 3) and the length distribution of contigs was displayed in Figure 1C. The obtained contigs were compared with the virus database. After comparison and screening, a total of 25 confirmed viral sequences and 520 suspected viral sequences were found (Table 4). Meanwhile, the percentage of DNA and RNA viral sequences in the total number of confirmed sequences were 56% and 44%, respectively (Supplementary Table S4). On the other hand, the DNA and RNA viral sequences accounted for 46.73% and 53.27% of the total suspected sequences, respectively (Supplementary Table S4). Moreover, the ratio of *Phages* in the confirmed and suspected viruses were 56.00% and 46.73%, respectively (Table 5). However, the identified sequence based on the comparison of the virus database existed some defects, because this method can only identify known viruses and the results may be false positives. Therefore, the variety of databases were increased to further identify viral sequences. Eventually, a total of 42 new viral sequences were identified (Figure 1D). Additionally, *Myoviridae* and *Herpesviridae* possessed the highest contig number at the family level (Figure 2A). The results of virus abundance statistics indicated that Nimaviridae and Whispovirus possessed the highest RPKM values at the family and genus levels, respectively (Figures 2B,C).

**Table 3:** The information of the obtained contigs.

**Sample**	**Total_base(Mb)**	**Total_num**	**Max_len**	**Min_len**	**N50**	**GC (%)**
YFP	6.27	10,600	5,879	301	577	50.47

Total_num the number of contigs. Max_lenthe: maximum length of contig; Min_len: the minimum length of contig.

**Table 4:** Statistical analysis of the viral sequence.

**Type**	**Total_base(Mb)**	**Total_num**	**Max_len**	**Min_len**	**N50**	**GC (%)**
Virus.confirmed	0.03	25	3,963	536	1,253	52.07
Virus.suspected	0.03	520	3,031	355	624	49.61

Total_num: total number; Max_len: maximum length; Min_len: minimum length.

**Table 5:** Statistical analysis of viral types in the confirmed and suspected virus.

**Type**	**Total**	**Phages (%)**	**Other_virus (%)**
Virus.confirmed	25	14 (56.00%)	11 (44.00%)
Virus.suspected	520	243 (46.73%)	277 (53.27%)

### Gene prediction and functional analysis

The contigs of virus were genetically predicted using MetaGeneMark (v3.38) software and the sequences of the gene nucleic acid <150 bp was filtered. The results revealed that a total of 52 genes were predicted (Table 6). The protein sequences of genes were compared with the virus sequence of UniProtKB/Swiss-Prot database by using blastp (v2.9.0+) to obtain the functional information of the virus. The results indicated that these genes were closely related to biological process, cellular component, molecular function and disease (Figure 2D).

**Table 6:** Statistical analysis of gene prediction results.

**Sample**	**Total_base (Mb)**	**Total_num**	**Max_len**	**Min_len**	**N50**	**GC (%)**
Viral.contig.gene_nucl	0.03	52	1,275	162	555	48.46

Total_num: total number; Max_len: maximum length; Min_len: minimum length.

## Discussion

Viral diseases are one of the greatest health challenges in the contemporary world, posing a huge threat to animal and public health (18, 19). Multiple factors regarding the viral emergence e.g., the climate, population growth, changing diets, human activity and environmental pollution have been demonstrated (20). These factors affect the spread of viruses in humans and animals and may accelerate the emergence of viral epidemics. Consequently, an in-depth investigation of viral diversity is essential to have a base line for the prevention and development of drug strategies against these diseases. Traditional identification methods have certain restrictions mostly based on the lack of corresponding cell lines, antibodies, gene sequences and biosafety facilities (21–24). Metagenomics provide a modern alternative in identifying and discovering novel and existing microorganisms especially in the samples which was not possible to analyze previously (25). Despite, non-human and environmental samples would need further confirmation and databases (26, 27). Theoretically, metagenomics techniques can perform the genomic characterization and identification of most microorganisms contained in the samples with a generic lab procedure (28). With the popularization and application of metagenomics, the new viruses in mammals have been observed gradually (29, 30).

Bacteriophages are potentially the largest source of gene diversity and are the most abundant biological entities in the ecosphere as well as marine environment (25, 31, 32). Previous research has indicated that temperate phages remain in the host in a latent state, whereas pathogenic phages can infect the bacterium of host and exit the cell by lysis or budding (33). Our results revealed a high abundance of phages in the Yangtze finless porpoise, which was consistent with the previous findings in other animals (34). This may be due to phages which are normally present in higher abundance in marine waters (35). Herpes virus is a DNA virus with envelope and can be divided into four subfamilies (36). Previous studies revealed that herpes virus can infect multiple livestock, poultry and fish and damage their skin, mucosa and nervous tissue (37, 38). Notably, cyprinids are the most susceptible to the herpes virus among aquatic animals. Previous research demonstrated that cyprinids were one of the main foods for the Yangtze finless porpoise (39–43). Moreover, Pei et al. also found herpes virus in the liver of dead Yangtze finless porpoise, which was consistent with our results (44). Therefore, further research is needed to determine whether the Yangtze finless porpoise can infect the herpes virus through the food chain.

The *Poxviridae* is a type of DNA virus with the largest virions generally brick-shaped or elliptic (45). The *Poxviridae* composed of *Orthpoxvirus, Parapoxvirus* and *Vaccinia* (46). Previous research revealed that members of the family *Poxviridae* posed a serious threat to the health of pig, poultry, rabbit and ruminant and resulted in skin damage (47). Furthermore, *Poxvirus* can also infect marine mammals such as whales, dolphins, seals, causing ring-shaped, pinhole or tattoo-like skin injury (48).

Retroviruses are spherical RNA viruses with envelopes, including *Tumorigenic virus, Lenti virus* and *Foamy virus* (49). Although most members of the *Retroviridae* cannot directly result in diseases, they could induce cancer growth (50, 51). It was reported that *Retroviruses* mainly infect humans and can be transmitted through contaminated water (52). Currently, the water quality of the Yangtze River has seriously degraded due to the discharge of domestic sewage, industrial and agricultural waste (53, 54). However, whether *Retrovirus* could infect Yangtze finless porpoise through polluted water sources remains to be studied.

Early studies indicated that the members of the *Arenaviridae* can cause hemorrhage, meningoencephalitis, hemorrhagic fever and neurological diseases in animal and humans (55, 56). It is still unclear whether members of the family *Arenaviridae* can result in the diseases of Yangtze finless porpoise. *Alphavirus* consists of 27 viruses, all of which are transmitted by vector insects including mosquito, louse, bug and mite (57, 58). The most members of *Alphavirus* can naturally infect birds and rodents (59). Moreover, there have been reports of *Alphavirus* isolated from amphibians and reptiles (60, 61). *Alphavirus* can cause fever, rash, arthralgia and other symptoms in humans and animals and can even lead to death in severe cases (62, 63). Notably, *Alphavirus* was also found in the Yangtze finless porpoise through viral metagenome.

In summary, this study first revealed the viral diversity of the Yangtze finless porpoise by metagenomics. Results indicated that there were many viruses in the Yangtze finless porpoise, such as *Arenaviridae, Ackermannviridae* and *Siphoviridae*. Moreover, most of the viruses were reported for the first time in the Yangtze finless porpoise. However, the viral diversity can be influenced by multiple external and internal factors, such as individual biological difference, virus degradation. Considering the restriction of a small number of samples and experiment conditions, we cannot completely remove all the above-mentioned influencing factors. However, our research first attempted to reveal the viral diversity of the Yangtze finless porpoise and supplemented the gap in its virus information. Additionally, our study may provide a theoretical basis for further research on the pathogenic mechanism and prevention and treatment of viral diseases to understand the natural flora in these animals by the use of advanced techniques to know the basis and the possibility of diagnosis and therapy of infectious diseases in these animals.

## Data availability statement

The original contributions presented in the study are included in the article/Supplementary material, further inquiries can be directed to the corresponding author/s.

## Ethics statement

The animal study was reviewed and approved by Ethics Committee of the Anqing Normal University.

## Author contributions

ZL conceived and designed the experiment data curation. ZL and KL contributed sample collection and reagents preparation. MH, FA, ZL, XD, HY, XC, ST, YZ, WL, BD, AZ, JZ, ZQ, HA, and DY revised the manuscript. All the authors reviewed the manuscript. All authors contributed to the article and approved the submitted version.

## Funding

This work was supported by the Hubei Yangtze River Ecological Protection Foundation-Dolphin-Vietnam Project, Anqing Normal University High-level Talent Introduction Program (2021), Aquatic Organism Protection and Water Ecological Restoration Project for External Development of Engineering Technology Research Center of Anhui Province Colleges and Universities (2021), and the key project of the Natural Science Foundation of Universities in Anhui Province (KJ2019A0551).

## Conflict of interest

The authors declare that the research was conducted in the absence of any commercial or financial relationships that could be construed as a potential conflict of interest.

## Publisher's note

All claims expressed in this article are solely those of the authors and do not necessarily represent those of their affiliated organizations, or those of the publisher, the editors and the reviewers. Any product that may be evaluated in this article, or claim that may be made by its manufacturer, is not guaranteed or endorsed by the publisher.
