# Supplementary material for: A metagenomic insight into the Yangtze finless porpoise virome
Source: Front Vet Sci. 2022 Sep 2;9:922623. doi: 10.3389/fvets.2022.922623 (PMC9478467; doi:10.3389/fvets.2022.922623)
Supplement: Supplementary file 1 [file Table_1.docx]

**Supplementary Table S1.** Statistical analysis of the viral sequence.

| Sample | Clean_reads (PE) | Virus_reads (PE) | Virus_percent (%) |
| --- | --- | --- | --- |
| YFP | 12686252 | 2172 | 0.0171 |
