# Supplementary material for: A metagenomic insight into the Yangtze finless porpoise virome
Source: Front Vet Sci. 2022 Sep 2;9:922623. doi: 10.3389/fvets.2022.922623 (PMC9478467; doi:10.3389/fvets.2022.922623)
Supplement: Supplementary file 2 [file Table_2.docx]

**Supplementary Table S2.** Statistical analysis of viral types.

| Sample | Other_virus (%) | Phages (%) |
| --- | --- | --- |
| YFP | 37.06 | 62.94 |
