# Supplementary material for: A metagenomic insight into the Yangtze finless porpoise virome
Source: Front Vet Sci. 2022 Sep 2;9:922623. doi: 10.3389/fvets.2022.922623 (PMC9478467; doi:10.3389/fvets.2022.922623)
Supplement: Supplementary file 3 [file Table_3.docx]

**Supplementary Table S3.**  The proportion of RNA and DNA virus.

| Sample | DNA virus (%) | RNA virus (%) |
| --- | --- | --- |
| YFP | 69.75 | 30.25 |
