# Supplementary material for: A metagenomic insight into the Yangtze finless porpoise virome
Source: Front Vet Sci. 2022 Sep 2;9:922623. doi: 10.3389/fvets.2022.922623 (PMC9478467; doi:10.3389/fvets.2022.922623)
Supplement: Supplementary file 4 [file Table_4.docx]

**Supplementary Table S4.** The ratio of RNA and DNA virus in the confirmed and suspected virus.

| Type | Total | DNA (%) | RNA (%) |
| --- | --- | --- | --- |
| virus.confirmed | 25 | 17 (68.00%) | 8 (32.00%) |
| virus.suspected | 520 | 429 (82.50%) | 91 (17.50%) |
